# Design and implementation of an asynchronous online course-based undergraduate research experience (CURE) in computational genomics

**DOI:** 10.1371/journal.pcbi.1012384

**Published:** 2024-09-12

**Authors:** Seema B. Plaisier, Danielle O. Alarid, Joelle A. Denning, Sara E. Brownell, Kenneth H. Buetow, Katelyn M. Cooper, Melissa A. Wilson

**Affiliations:** 1 School of Life Sciences, Arizona State University, Tempe, Arizona, United States of America; 2 Center for Evolution and Medicine, Arizona State University, Tempe, Arizona, United States of America; 3 Research for Inclusive STEM Education Center, Arizona State University, Tempe, Arizona, United States of America; SIB Swiss Institute of Bioinformatics, SWITZERLAND

## Abstract

As genomics technologies advance, there is a growing demand for computational biologists trained for genomics analysis but instructors face significant hurdles in providing formal training in computer programming, statistics, and genomics to biology students. Fully online learners represent a significant and growing community that can contribute to meet this need, but they are frequently excluded from valuable research opportunities which mostly do not offer the flexibility they need. To address these opportunity gaps, we developed an asynchronous course-based undergraduate research experience (CURE) for computational genomics specifically for fully online biology students. We generated custom learning materials and leveraged remotely accessible computational tools to address 2 novel research questions over 2 iterations of the genomics CURE, one testing bioinformatics approaches and one mining cancer genomics data. Here, we present how the instructional team distributed analysis needed to address these questions between students over a 7.5-week CURE and provided concurrent training in biology and statistics, computer programming, and professional development. Scores from identical learning assessments administered before and after completion of each CURE showed significant learning gains across biology and coding course objectives. Open-response progress reports were submitted weekly and identified self-reported adaptive coping strategies for challenges encountered throughout the course. Progress reports identified problems that could be resolved through collaboration with instructors and peers via messaging platforms and virtual meetings. We implemented asynchronous communication using the Slack messaging platform and an asynchronous journal club where students discussed relevant publications using the Perusall social annotation platform. The online genomics CURE resulted in unanticipated positive outcomes, including students voluntarily discussing plans to continue research after the course. These outcomes underscore the effectiveness of this genomics CURE for scientific training, recruitment and student-mentor relationships, and student successes. Asynchronous genomics CUREs can contribute to a more skilled, diverse, and inclusive workforce for the advancement of biomedical science.

## Author summary

As technology advances, there is a growing demand for research scientists trained in computational biology but it can be difficult to introduce computer programming and statistics to biology students. One way to meet this demand in an inclusive way is to provide more research opportunities for online students, a significant and growing community which includes many groups underrepresented in the science workforce. We present a course designed for fully online undergraduate biology students where they can work asynchronously to address a novel research question. We show how we divided research projects among the students of the class, leveraged remotely accessible computational tools and online messaging platforms, and created custom learning materials and assessments to teach the students the necessary biology, computer programming, and communication skills needed for each research project. We demonstrate that students were able to learn the course objectives and cope with academic stresses. Research can be designed around questions in many topics, so we hope that our design can help others to create remote computational research courses in their field.

## Introduction

Biomedical science has seen enormous growth in the amount of genomic data produced to investigate the molecular underpinnings of cell biology in health and disease. As high-throughput molecular assays and technology for data processing and machine learning advance, there is an increasing need for cross-disciplinary computational analysts trained to understand biology, genetics, statistics, pharmacology, and mathematical modeling. While typical undergraduate biology courses provide students with a broad background in molecular biology, genetics, and chemistry, many programs still lack substantial instruction in the computation and quantitative analysis [1–3] necessary to analyze genomics data. Barriers to integrating computation include difficulty finding instructors who have had formal training themselves [3,4], so having examples of course materials and techniques for teaching computational genomics and bioinformatics would be beneficial for the genomics research community. In this manuscript, we describe a course format that can be used to successfully teach computational genomics analysis to biology students in a fully asynchronous, online research environment in order to broaden access to research training.

Course-based undergraduate research experiences (CUREs) are formal courses in which students use well-established scientific practices to participate in novel research projects of interest to the broader scientific community [5]. CURE topics are often driven by questions that arise in the instructing faculty’s area of interest, thereby providing students with mentors and skills as well as providing mentors with a pipeline to train and recruit students that can advance research programs [6]. CURE research projects are aimed at publication, which allows both mentors and students to contribute to the field while allowing the students to gain key research skills and build a science identity [7].

We designed a course-based research experience (CURE) specifically for fully online undergraduate students in biology. Online undergraduates are more likely to be from underrepresented demographics in science, including first-generation college students, women, low-income households, and nontraditional adult learners [8,9]. Online research experiences have the potential to increase the amount of historically underrepresented student participation in STEM (Science, Technology, Engineering, and Math), increase their application to (and likely enrollment into) graduate programs, and open additional career opportunities, thereby making access to STEM education more equitable and the future workforce more diverse [10–12].

Bioinformatics and computational biology represent a unique opportunity for the development of fully online CUREs. First, computational analysis resources for -omics (e.g., transcriptomics, genomics, proteomics) level studies are often hosted on high-performance biocomputing clusters, cloud computing environments, or web-based computational analysis platforms whereby users may log in from any location as long as they have a computer and sufficient internet access. Second, transitioning into computational research can be especially challenging for students, perhaps even more so in an asynchronous setting [13,14], but engaging with bioinformatics in a CURE setting can give students more detailed instruction on how to address common sources of anxiety. For biology students who may not have anticipated learning computational skills, bioinformatics research can include anxiety about a lack of pertinent background knowledge, computer programming anxiety and inexperience, and issues related to accessibility and inclusion in the virtual classroom [13,15]. While these factors have been linked to high attrition rates in online STEM courses, the literature shows that retention can increase with student-specific interventions [16], which we aimed to incorporate in this CURE.

In this manuscript, we describe how we developed and implemented an online CURE in computational genomics to study 2 different research questions over 2 iterations of the course. We discuss how the required analysis was distributed among students in an asynchronous format over 7 week-long modules and how research-specific learning materials were designed to promote student success. We demonstrate that our implementation of this CURE led to learning gains among the class and report student responses to research and coding. This presentation of how a computational genomics CURE was designed and implemented for online students is intended to serve as a template for others seeking to expand inclusive and accessible research opportunities at their educational institutions.

## Course design and implementation

### Ethics statement

Before students began the online genomics CURE, formal written consent was obtained as part of a student experience and demographics survey. This project was conducted with an approved protocol through the Arizona State University Institutional Review Board (approval number STUDY00013025). Students were asked if they were at least 18 years old and if they consent to be part of this study. All students that consented to the study indicated that English was their primary language.

### Prerequisites and course format for online biology students

Students enrolled in this course were online students from a variety of biology majors (Biological/Biomedical Sciences, Neuroscience, Biochemistry, Conservation Biology, and Ecology), mostly in the final 2 years of their undergraduate degree program. Enrollees had interest in computational biology, but most had very little computer programming experience and many had not had opportunities to do research during their online degree program. To give students a basic foundation for computational analysis, students were required to take a prerequisite 7.5-week course in the first half of the semester (BIO 439: Computing for Research; session A in Fall 2022 and 2023). This course assumed no prior coding experience; students were introduced to command line programming, bash scripting, navigating a high-performance computing cluster, and ran through basic genomics analysis (fastq file quality control, alignment, and variant calling). The assignments in the prerequisite course are meant to be a broad first look at how students respond to computer programming and are not specific to the research project chosen for the CURE. All students were given the option of continuing on to the online genomics CURE (session B in Fall 2022 and 2023) but students needed to get approval from the lead instructor before being allowed to continue into the research session.

### Research questions for each CURE

**Fig 1:**
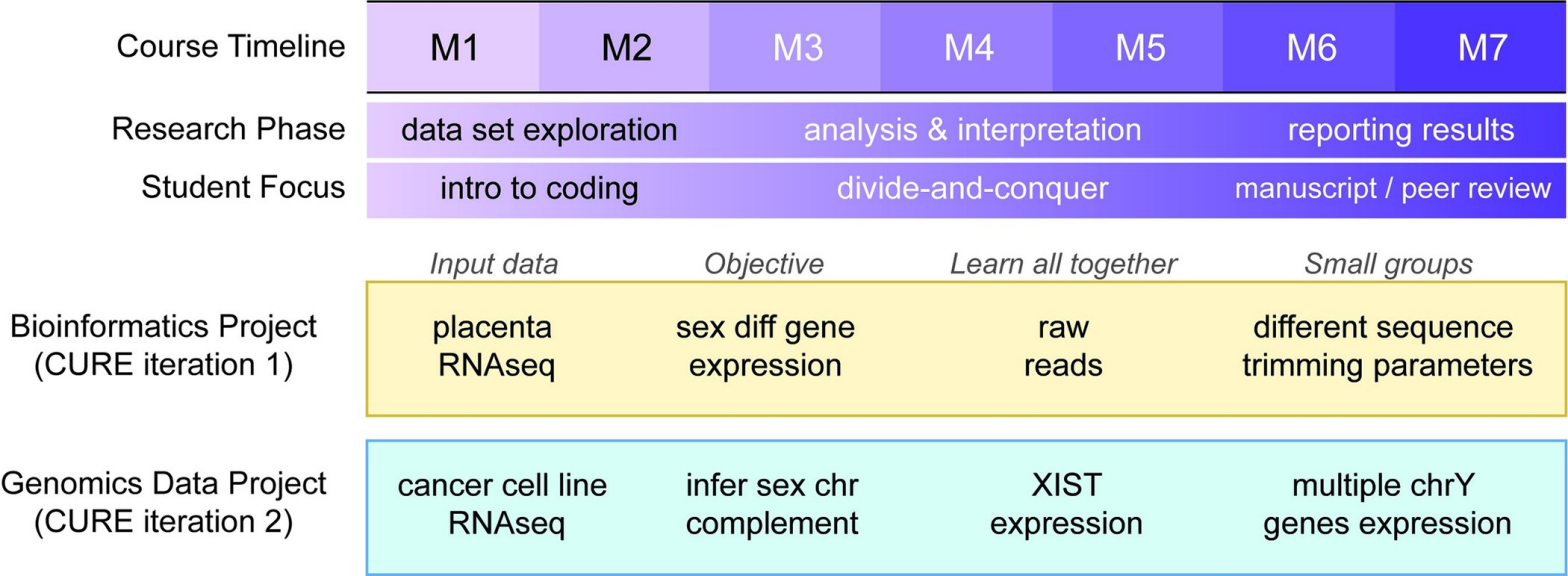
Project summaries and distribution of analysis among students. Two iterations of the online genomics CURE were taught in the same format and used to conduct 2 completely different research projects. The first 2 modules were dedicated to exploration of the data set being analyzed and featured an introduction to the coding language being used in the course. The middle modules were used to divide the required analysis between the students of the class. For iteration 1, students studied how parameters for quality trimming of RNA sequencing data affected identification of genes differentially expressed by sex in the human placenta. For iteration 2, students used expression of specific sex chromosome genes to infer the sex chromosome complement in cell lines used as models for human cancer. In the last modules, each student described the results of the study in a manuscript which was peer reviewed by other students.

We have implemented 2 iterations of the online asynchronous genomics CURE covering 2 separate research questions (**Fig 1**, details in **S1 Methods**). The pilot iteration of the CURE was based on a bioinformatics project that investigated the effect of sequencing quality on inference of sex differences in gene expression in human placenta. Placenta gene expression data were processed with a range of parameters for sequence quality trimming software and students were asked to compare results to determine if the list of sex differentially expressed genes changed due to the stringency of the parameters chosen. The second iteration of CURE was based on inferring the sex chromosome complement in human cancer cell lines based on expression of genes on the sex chromosomes. Both of these projects were developed organically in the research laboratory of the course instructors, thus ensuring that the instructors had the required expertise to lead the course research and aiding the instructors in communicating why and how the research would contribute to the field.

### Distribution of analysis among students

While research training is typically conducted with a one-to-one mentor–student relationship, we employed a paradigm wherein a small team of instructors can provide mentorship to a larger group of students and simultaneously facilitate the development of a network of peers (**Fig 1**). During the first 2 modules, course materials and course communications were focused on getting all the students in the class the background information needed to understand the research aims and the way that the research is to be conducted. This included tutorials in the R programming environment, learning how the data were generated, and learning the biological, genomic, and statistical concepts necessary to understand the research aims. In Module 3, all students were assigned the same initial analysis by modifying template code provided by the instructors to facilitate troubleshooting coding errors and streamline guidance on how to interpret the results. For Module 4, students were split into smaller working groups to adapt the code to analyze different but related data sets that address the research question. Instructors assigned student working groups, putting communicative students that were showing technical skills with students that were struggling to maximize the likelihood that all parts of the divided work would be completed successfully and that students had the opportunity to learn from each other. Given small group assignments, students could have someone to work with if they desired or could just as easily work independently; students assigned to the same group could directly compare results and troubleshoot together if needed. Following Module 4, instructors checked data files generated by students for errors and put all the results from all student groups in a shared online location. For Module 5, students were asked to plot and interpret trends across the full data set. This module allowed struggling students time to catch up if they had coding issues in the previous modules and students that were ahead to expand on the analysis they had completed. In Module 6, each student was asked to present their results in the format of a scientific manuscript. Students were allowed to learn from the writing of the other students by conducting a peer review in Module 7. Students were also asked to turn in all code, figures, and output data files so that they can be used to prepare for publication after the completion of the course. In this way, we distributed the analysis, interpretation, and description of the research question equally among the students while simultaneously building in redundancy to make the research goals more robust to individual student challenges.

## Translating research plan into learning materials

### Backwards design of learning objectives

**Fig 2:**
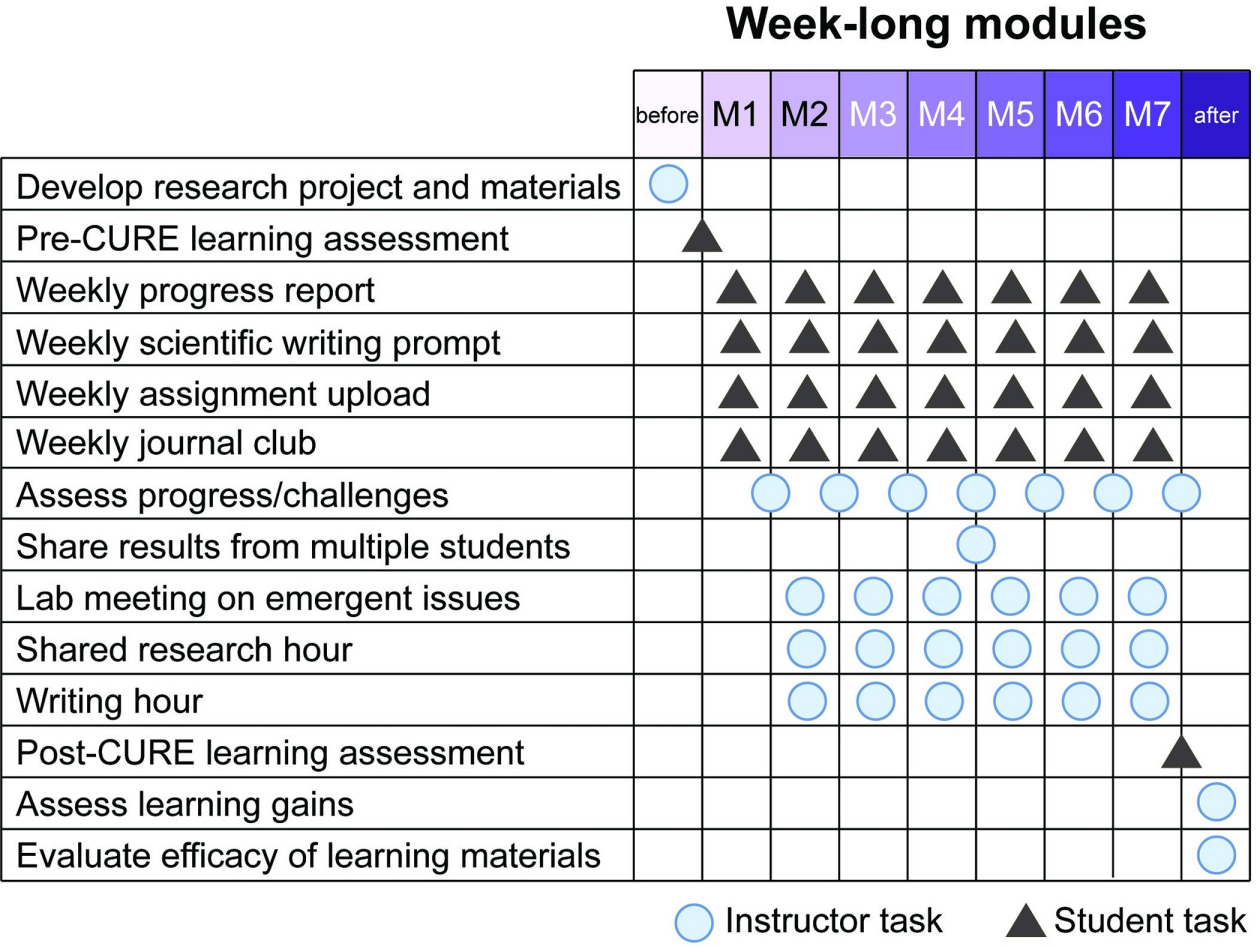
Instructor and student contributions over 7-week CURE. Instructor contributions are labeled as circles, student contributions in triangles. Students began by filling out a pre-assessment to show their baseline level of knowledge on the research topic. Each week, students completed research goals and turned them in as assignments along with a progress report communicating their achievements and challenges. Students were assigned weekly scientific writing prompts and publications to read in a journal club designed to help them slowly build up their final reports in Module 6. Instructors used all of these submissions to select topics for weekly lab meetings conducted using teleconference software and recorded for students that could not attend. Instructors hosted shared research (office) hours throughout the week. Instructors monitored progress on research goals and distributed data generated by individual students at the midpoint of the CURE. Following the completion of the course, students completed a learning assessment which instructors analyzed to see which areas students were able to increase their knowledge in as well as misconceptions and struggles that could be avoided in future CUREs.

Learning objectives were written to match the knowledge and skills needed to perform each project within the seven-module format. The main objective for each iteration of the CURE was based on the research project chosen (**Fig 1**) and then mapped over 7 modules (listed in GitHub repository (https://github.com/SexChrLab/CURES)). Once the learning objectives were mapped out across the 7 modules, module learning pages, reading assignments, and coding assignments were developed to guide the students in achieving those learning objectives. Retention of the most important information and learning of skills in each module was tested using questions in learning assessments (quizzes) during each module. Project descriptions and matching learning objectives for both iterations of the course are available in our **S1 Methods** and GitHub repository (https://github.com/SexChrLab/CURES). Instructors analyzed weekly progress reports and assessments to address topics that needed clarification, common misconceptions, and ways to support student learning success in real time (**Fig 2**).

### Module learning pages

In lieu of expensive textbooks and akin to lab-based research projects, the instruction team wrote collaboratively to produce freely available reference materials to guide the students as they performed the analysis for the chosen research project. Each module had pages posted in the Canvas learning management system. In an introductory module (Module 0), students were introduced to the overall format of the course, introduced to the idea that they will be doing real research where they will collaborate to discover something new, and encouraged to engage in course communications. For Modules 1 to 7, learning pages were written in a modular fashion to keep the format of each module consistent so students could follow them more easily and to increase reusability for future CURE projects. Each learning page had 3 sections: (1) Biology/Statistics; (2) Coding; and (3) Professional Development. The Biology/Statistics section was used to describe biological relevance to the analysis, statistics necessary to test hypotheses being generated using the data available, and explain ways to interpret results. The Coding section featured formal instruction on computer programming and explained aspects of the code provided by the instructors. The Professional Development section showed the students ways to seek out information from the literature, described the publication and peer review process, and the cultural norms of scientific research. Additional learning resources for novices and enrichment for more advanced students were included as “Additional Resources.” Published bioinformatics workflows, vignettes, and tutorials were incorporated and/or modified as needed to demonstrate analysis relevant to answering the research question. Module learning pages for both iterations of the course are available in our GitHub repository (https://github.com/SexChrLab/CURES).

Writing learning pages for CUREs took less time with each iteration. For the first iteration, developing the course materials took about 1 to 2 weeks per module because the instructors developed all learning materials and assessments completely from scratch while simultaneously deciding on the overall framework of the course. However, developing materials for the second iteration was much quicker (about 3 days per module) because the overall framework for the course was already created and sections of the learning materials could be reused. The Professional Development sections were written to be evergreen and many of the Coding sections could be reused because both iterations of the CURE used code written in the R programming language. As the research being conducted in the CURE is based on the specialty of the instructors, we anticipate that instructors of genomics CUREs will build their own library of learning materials that can be reused, repurposed, or shared with other students for training outside the CURE from year to year.

### Template code

To facilitate implementing functional code in a short timeline, the instruction team developed template code that the students were asked to modify and build on to complete the analysis required for the research project. Before the course started, instructors processed and prepared the starting input data. For Iteration 1 of the CURE, students were given template code to perform differential gene expression analysis that they could modify to run with different data sets. For Iteration 2, template code was provided to plot expression of a specific gene in cancer cell lines that they could modify to plot other genes and features of interest. Template code was provided in RMarkdown format so that code, products of code, and descriptions of code could be easily created in a single document used for grading, communication, and publication. Template code had many descriptive comments to explain each computational step and model best practices for coding technique. Tutorials assigned at the beginning of the CURE were chosen based on how well they explained the features in the template code so that students would feel more confident in making changes to suit the purposes of the research. Template code for each iteration of the course is provided in Github (https://github.com/SexChrLab/CURES).

### Student assessments and surveys

#### Pre- and post-learning assessments

Students took an identical learning assessment before and after completing the genomics CURE (**Fig 2**). Questions were designed by the instructors to match the learning objectives of the course in Biology/Statistics, Coding, and Professional Development, and vetted by outside genomics experts. Likert surveys were included to gauge student comfort levels with specific skills taught in the genomics CURE. Scores and answers were not revealed to the students and students received full credit regardless of the percentage of correct answers.

#### Weekly progress reports

To maintain an authentic research experience, the instruction team developed an open-response summative assessment for students to submit weekly, designed in the fashion of progress reports submitted in the lead faculty instructor’s laboratory (**Fig 2**). To focus the students’ attention on research progress (not just grades on assessments), the progress report prompted students to list their accomplishments, challenges they faced, and how they addressed those challenges. The second iteration of the CURE also included a scientific writing prompt, wherein students were asked to describe specific methods and results each week, receiving iterative feedback prior to compiling their final manuscript report.

#### Coding assignments

Research milestones were assessed using weekly coding assignments, typically requiring upload of code reports and data output files. Feedback was provided to students through the Canvas learning environment using a custom rubric highlighting key concepts. Because more value was placed on effort and progress than on specific outcomes, the progress report was worth 100 out of 125 points of the weekly module assignment grade and coding assignments were only worth 25 points.

### Manuscript and peer review

As a final project in the CURE, students were asked to write a report in the format of a manuscript for publication and then asked to conduct peer reviews on manuscripts written by their classmates (**Fig 2**). This trained students to communicate scientific results with the level of accuracy and detail expected at a professional level. The professional development section of several modules was used to walk students through how to put together the various parts of their manuscript. Students were taught how to write detailed legends for figures produced as the research project was conducted in Modules 3, 4, and 5, then asked to create a storyboard outline of these figures to weave a complete set of related analyses that address the research project aims in Module 5, before fully writing about these results in Module 7. Examples of how to keep track of and document software packages used in code in the methods section of the manuscript were featured in all template code. Class discussions on topics involved in the research helped develop context that was featured in the abstract, introduction, and discussion portions of the manuscript. Many students wrote about results that were unexpected as those results fueled many discussions in lab meetings and Slack throughout the course, such as having a lot of overlap in results with different trimming parameters in Iteration 1 and cancer cell lines having sex chromosome gene expression that did not match what was expected based on the reported sex of the patient from which the cancer cell line was derived in Iteration 2. As all students did similar analyses, they were able to appreciate the descriptions and insights offered by their classmates during the peer review process. The rubric used to grade peer reviews of manuscripts is in the provided Github repository (https://github.com/SexChrLab/CURES).

### Class communication

The primary mode of communication with students was the Slack messaging system so that responses to questions and posts were visible to all students. This platform was integrated into the Arizona State University Canvas learning management system and allowed for asynchronous communication between students (see **S1 Methods** for details and alternatives). Students were encouraged to post and respond to Slack messages about confusing concepts, coding problems, and any other challenges encountered during the course. Instructors posted about common misconceptions, bug fixes, activities and resources for learning enrichment, and encouragement for students as they struggle with challenging material. In addition to Slack, students were given several opportunities each week to meet with the instruction team synchronously: lab meeting, writing hours, and shared research hours (**Fig 2**). Optional weekly lab meetings recorded and transcribed in Zoom covered student-reported challenge topics. Optional weekly writing hours gave students the opportunity to discuss interpretation of findings they would write about in their weekly assignments and manuscript. Optional shared research hours were offered twice a week, giving students the opportunity to troubleshoot code with the instruction team live with options to share their screen.

### Asynchronous journal club

In the second iteration of the CURE, instructors implemented an asynchronous journal club to help students engage with peer-reviewed literature relevant to the research project (**Fig 2**). To do this, the instructors chose 1 publication each week for the first 5 modules for the students to read (leaving the last 2 modules with more time to focus on the manuscript and peer review). To encourage collaborative learning, the chosen publications were posted using the Perusall social annotation environment and students were given points toward their final grade for posting comments as they read the paper (see **S1 Methods** for more information). We found that the students pointed out what they thought was interesting or relevant about the publications and asked and answered each other’s questions about the journal articles. This format of journal club provided the flexibility needed for online student learning while providing asynchronous but meaningful discussions about important published works in the field.

### Grading and instructor communications

Custom rubrics and examples were provided to students to describe expectations for assignments and scientific writing. The majority of the course grade for the CURE was based on the weekly progress report so the majority of the grading efforts of the instructors were dedicated to this (**Fig 2**). As the progress report was divided into sections, instructors were able to split the work, typically with one instructor grading the accomplishments and challenges sections and another grading the scientific writing and coding assignments addressing the research aims. Instructors held a short weekly meeting to discuss research progress and how to address student challenges. Since a great deal of the grade for the CURE was based on open-response writing, instructors watched carefully for signs of academic dishonesty, including inappropriate use of artificial intelligence chatbot engines and copying between students (**S1 Methods**), but because so much emphasis was placed on progress over products, the vast majority of students submitted their own work and experiences throughout the CURE.

## Assessing student learning and student comfort

**Fig 3:**
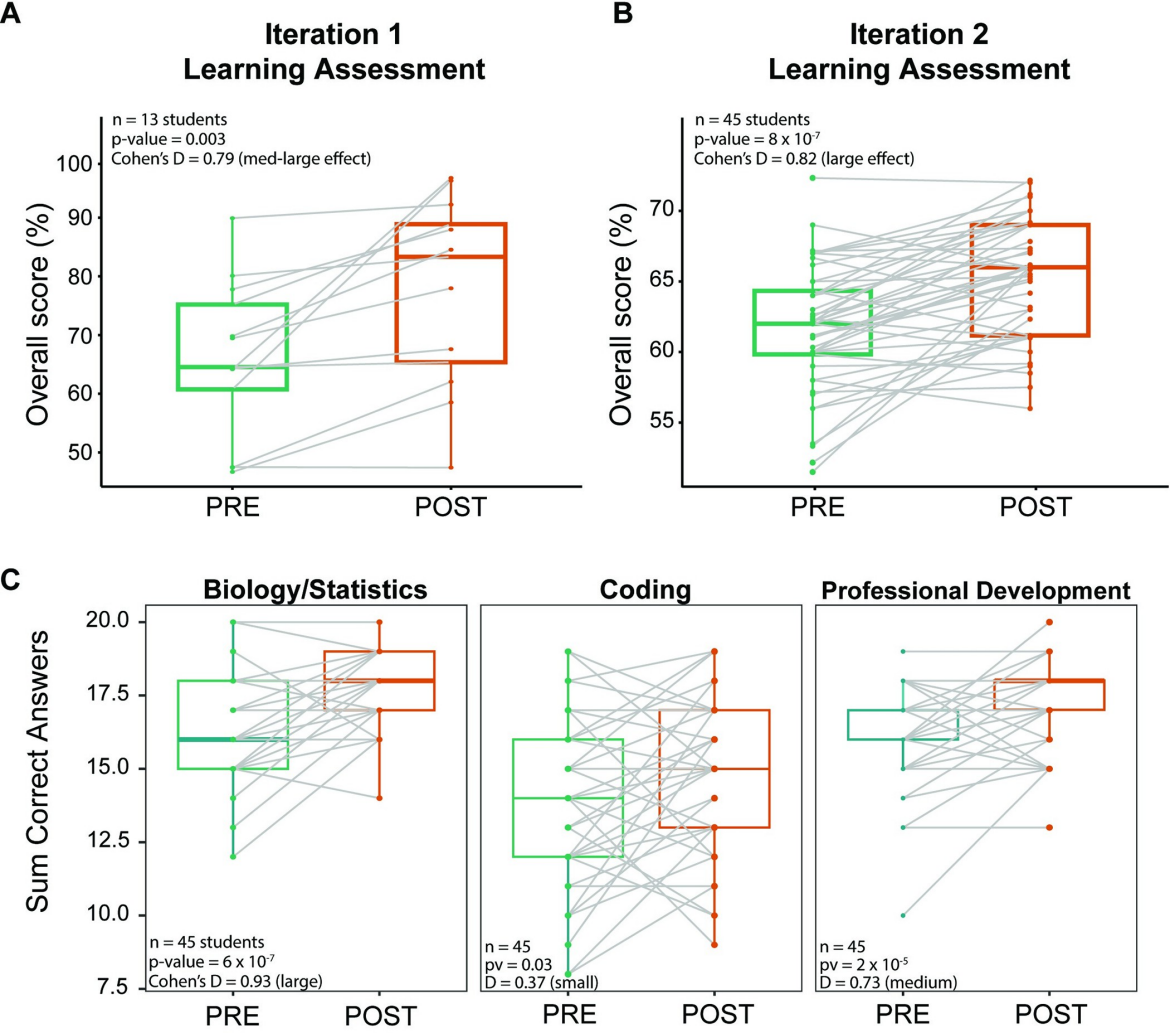
Increase in student knowledge and skills after genomics CURE. (A, B) Boxplots depicting mean student assessment scores before (green) and after (orange) completing the genomics CURE. Each point represents a student who completed both the pre- and post-assessments and the lines connect pre-assessment and post-assessment scores for each student. The mean class score significantly increased from 65.96% to 77.61% (paired *t* test *p*-value 0.003, Cohen’s D-statistic 0.79, medium-large effect) for iteration 1 (A) and 61.62% to 65.16% (paired *t* test *p*-value 0.00002, Cohen’s D-statistic 0.82, large effect) for iteration 2 (B). (C) Boxplots depicting each pre-assessment (green) and post-assessment (orange) score for all questions divided by topic for CURE Iteration 2: Biology/Statistics, Coding, and Professional Development.

A significant improvement in overall scores between pre- and post-assessments was observed in both iterations of the genomics CURE. The first iteration of the CURE showed a significant increase of 11.64% in the mean score from 65.96% to 77.61% (*n* = 13 students, *p* = 0.003, **Fig 3A**). This is considered to be a medium to large effect size as evaluated by Cohen’s d-statistic (d = 0.79, medium effect size range = 0.5–0.8, large effect size range >0.8). The second iteration of the CURE showed an increase of 3.5% in the mean score from 61.62% to 65.16% but this increase was more statistically significant and deemed a large effect size given a much larger class size (*n* = 45 students, *p* = 8 × 10^−7^, d = 0.82, **Fig 3B**). Students consistently showed the most significant increase in the Biology/Statistics section in both iterations of the CURE (**Figs 3C and S2**), while the Coding and Professional Development sections showed small to medium levels of effect on student learning. The learning assessment questions for the second iteration were also given a subtopic within Biology/Statistics, Coding, and Professional Development, which shows that the increased learning in Professional Development in the second iteration (**Fig 3C**) was driven by a significant improvement in the subsection about reading scientific papers (**S3 Fig**). This is likely due to the implementation of the asynchronous journal club specifically in Iteration 2 of the online genomics CURE. Progress reports for students whose learning assessment scores did not improve after taking the CURE included reports of unexpected issues in their personal lives as well as some who were unable to make the necessary learning gains required to understand and implement the code. Likert questions included on the learning assessment also showed increased comfort with the skills taught during the CURE for many students (**S4 Fig**).

## Understanding student experiences

**Fig 4:**
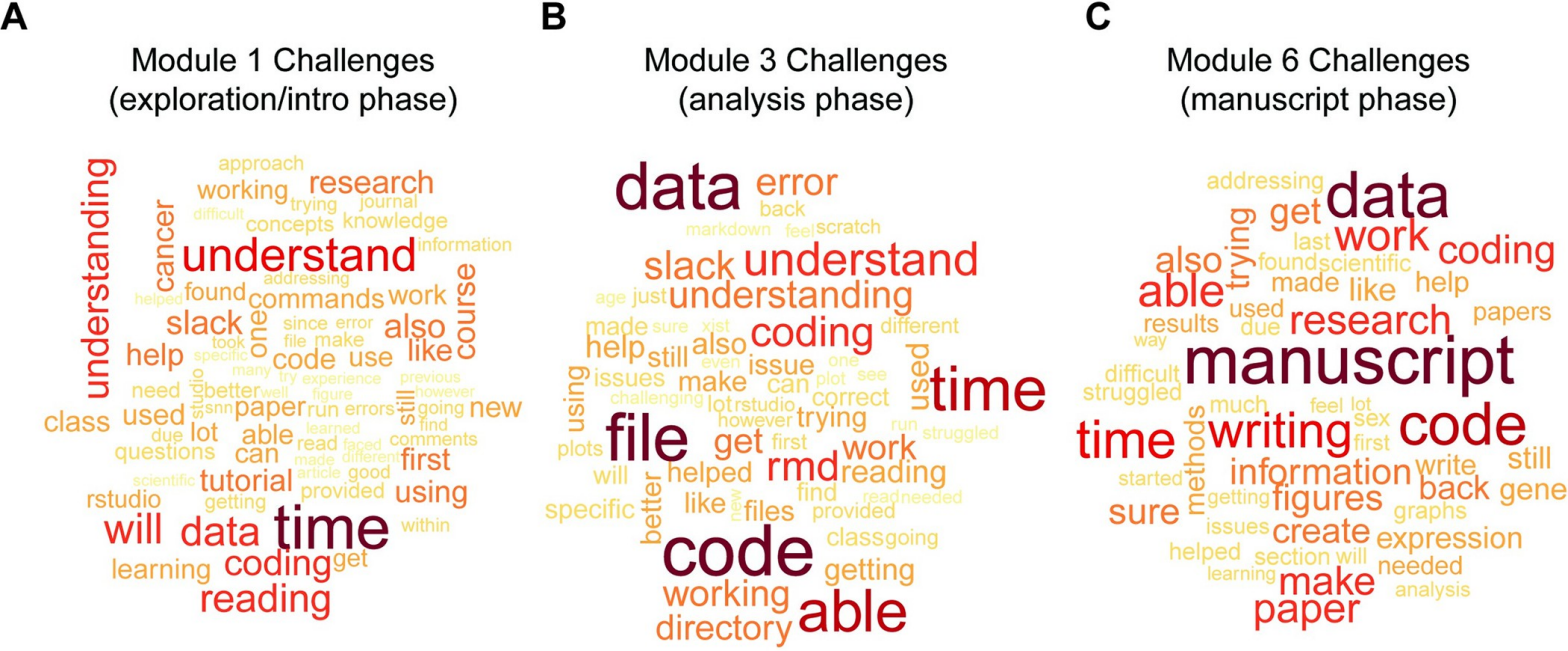
Challenges across CURE modules (Iteration 2). (A–C) Word cloud summary of challenges reported in weekly progress reports in 3 main research phases: Module 1 (A) representing the data exploration and coding introduction phase, Module 3 (B) representing the analysis and interpretation phase, and Module 6 (C) representing the reporting results phase. Word use frequency is shown by size and color (larger and darker shade of red for higher frequency).

To more thoroughly understand student experience throughout the CURE and increase student engagement, each week the instruction team reviewed the progress report for challenges reported and how the student responded to or coped with those challenges. Summaries of the progress reports (**Figs 4A–4C and S1**) show that students frequently felt challenged by needing to understand the material, writing and troubleshooting code, and managing their time to complete the research aims while having a full course load. Time was mentioned mostly frequently in the exploration and introduction phase (Modules 1 and 2) as it is challenging to jump into a research project; many students wrote about searching for more information to fuel their curiosity beyond the learning materials. In the analysis phase of the research (Modules 3, 4, and 5), coding became the most frequent reported challenge. Reading details about what aspect of the coding was the most challenging for students allowed the instructors to provide support to students to help solve common issues and to improve coding tutorials in future iterations of the CURE. Additionally, many students reported in their progress reports that they had full-time jobs and care-taking responsibilities that contributed to difficulty with time management. In response to time management concerns, instructors provided learning materials in smaller sections and provided time estimates where possible to help students manage their work time effectively. In the analysis modules, students commonly mentioned Slack, and asked for help understanding errors while compiling the R Markdown report, demonstrating that they looked to collaborate to solve coding problems. In the reporting or manuscript phase (Modules 6 and 7), students reported challenges with writing and getting information and figures into their manuscript. Many students reported that this was the first time they were asked to synthesize new results instead of applying techniques indicated by instructors to get a predetermined result. Some weren’t sure if they had done enough to address the research aims or if their writing was clear enough; instructors were able to provide feedback and resources to address these concerns. Many students reflected on the quality of their own work after reading other students’ work in the peer review in Module 7. Analysis of general trends on how students coped with challenges showed that a high proportion of students employed adaptive coping strategies (problem solving, support seeking, information seeking, and self-reliance) throughout the course as these were encouraged and rewarded, while the rate of maladaptive coping strategies reported increased towards the end of the course as students worked hard to describe results in their final manuscript and had struggled to keep up with final assessments while also managing the rest of their academic schedules (**S5 Fig**). Select statements from students’ progress reports can be found in the **S1 Methods**. These statements demonstrate that while students struggled trying to learn skills and knowledge they felt were interesting and important, ultimately, they were able to achieve meaningful growth in their research mindset and overall knowledge of the topics being studied. Progress reports provided a way for instructors to look beyond students’ grades to follow their journey through the research project while learning to manage the uncertainty that comes with doing research. Progress report helped instructors provide feedback and resources tailored to the needs of the students in each iteration of the CURE.

## Discussion and future directions

Upon completion of the genomics CURE, all students were invited to continue to work on the research project the following term. Many students requested to continue on with the project by doing follow-up analysis or present the genomics results for research symposiums and publication. Follow-up studies focused on assessing the generalizability of the results from the CURE with other related data sets and analytical tools. In progress reports and course discussions, students discussed seeking out research and professional development opportunities outside of the CURE research project. Additionally, mentors may be able to recruit students as more permanent members of their laboratory. This study demonstrates that students can be successful in online research experiences that incorporate accessible learning materials, multiple options for class communication, and open-response progress reports to monitor achievements, challenges, and coping strategies.

By directly assessing student learning and experiences throughout the CURE, the instructors were able to tell which areas of the course are effective and which to prioritize for improvement. Based on the feedback from students, instructors continually improved the introductory materials for the earlier modules in the course to help ease students into the research project and coding. Instructors are currently developing better ways to share communication and discussion between students including a bulletin board Slack channel where instructors can post results and accomplishments for all students to learn from throughout the course and provide more detailed templates for the final manuscript and other assignments to communicate expectations more clearly. Instructors are investigating more automated ways to collect and analyze information from the progress reports to make grading more scalable and inform discussion topics and interventions. An instructor handbook is being created to help share lessons learned over many iterations of the CURE.

Opening genomics research opportunities to online students using an asynchronous format allows many students who would typically be excluded to participate in important research endeavors. Asynchronous genomics CUREs are a way to bring valuable research opportunities, mentorship, and analytical skills to many students who can go on to contribute to a more diverse and capable workforce to tackle an ever-expanding set of genomics challenges.

## Supporting information

S1 Fig Summary of accomplishments and challenges section of the weekly progress reports for Iteration 2 of the CURE. For each module, word clouds are provided to summarize what students were accomplishing given the research aims (provided on the left in blue) and what challenges were encountered. Word size and color is used to highlight high frequency words, larger and dark shade of red indicating high frequency.
(TIF)

S2 Fig Increase in student knowledge and skills after genomics CURE by Topic (Iteration 1). Boxplots depicting each pre-assessment (green) and post-assessment (orange) scores for all questions divided by topic for CURE Iteration 1: Biology/Statistics, Coding, and Professional Development.
(TIF)

S3 Fig Increase in student knowledge and skills after genomics CURE by Subtopic (Iteration 2). Violin plots depicting each pre-assessment (green) and post-assessment (orange) scores for all questions divided by subtopic for CURE Iteration 2. Subtopics showing paired *t* test *p*-value less than 0.01 are highlighted in red, between 0.01 and 0.05 in orange, and between 0.05 and 0.1 in yellow.
(TIF)

S4 Fig Questions about student comfort level in specific skills in the genomics CURE from pre- and post-learning assessments. Students were asked to rate their comfort level on a scale from very uncomfortable to very comfortable (6 options total, responses assigned numerical values between −3 and +3) for skills used throughout the course: programming in R, reading and writing scientific papers, asking questions about coding in a class setting, and using command line programming in a Linux environment. Boxplots depicting each pre-assessment (green) and post-assessment (orange) scores for all 5 questions for all students are shown with a paired *t* test *p*-value showing the statistical significance of the improvement after the completion of the CURE.
(TIF)

S5 Fig Trends in coping strategies across CURE modules. Proportion of students reporting various coping strategies to overcome challenges encountered during genomics research. Responses for progress reports for each module were categorized as adaptive, maladaptive, or those that could be either depending on context. Adaptive themes include problem solving (red), support seeking (orange), information seeking (gold), self-reliance/emotional regulation (olive), and cognitive restructuring (green). Maladaptive themes include escape (light blue), isolation (blue), rumination (purple), helplessness (lilac), delegation (fuchsia), and opposition (pink).
(TIF)

S1 Methods This section provides additional details on the research projects chosen for both iterations of the genomics CURE, instructions given to instructors and students to promote a collaborative research environment, implementation of various tools used for instruction, and insights that could be helpful when creating a CURE. (DOCX)
