# Supplementary figures and images for: Design and implementation of an asynchronous online course-based undergraduate research experience (CURE) in computational genomics
Source: PLoS Comput Biol. 2024 Sep 12;20(9):e1012384. doi: 10.1371/journal.pcbi.1012384 (PMC11392228; doi:10.1371/journal.pcbi.1012384)

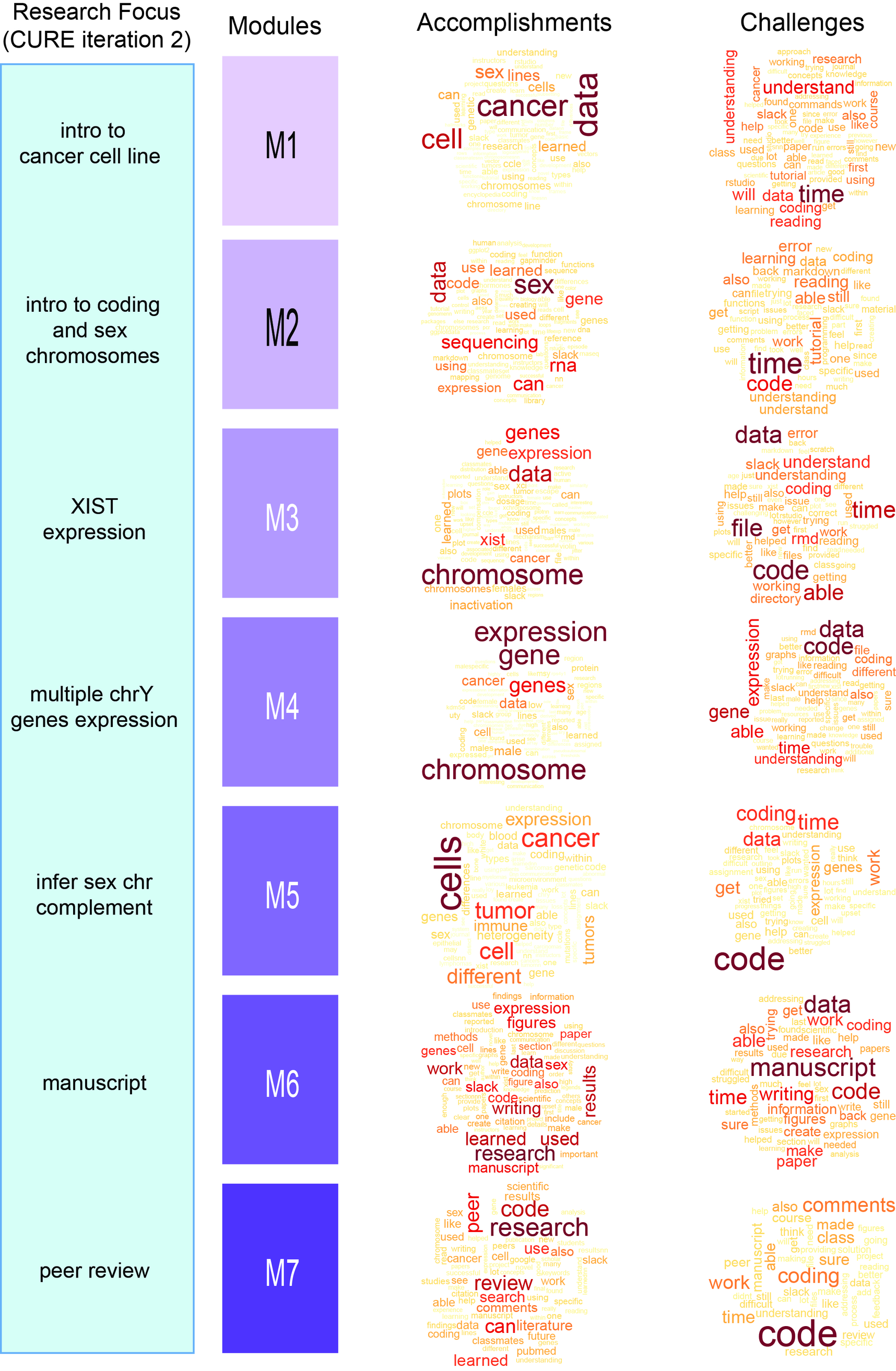

Supplement: S1 Fig — For each module, word clouds are provided to summarize what students were accomplishing given the research aims (provided on the left in blue) and what challenges were encountered. Word size and color is used to highlight high frequency words, larger and dark shade of red indicating high frequency. (TIF) [file pcbi.1012384.s001.tif]

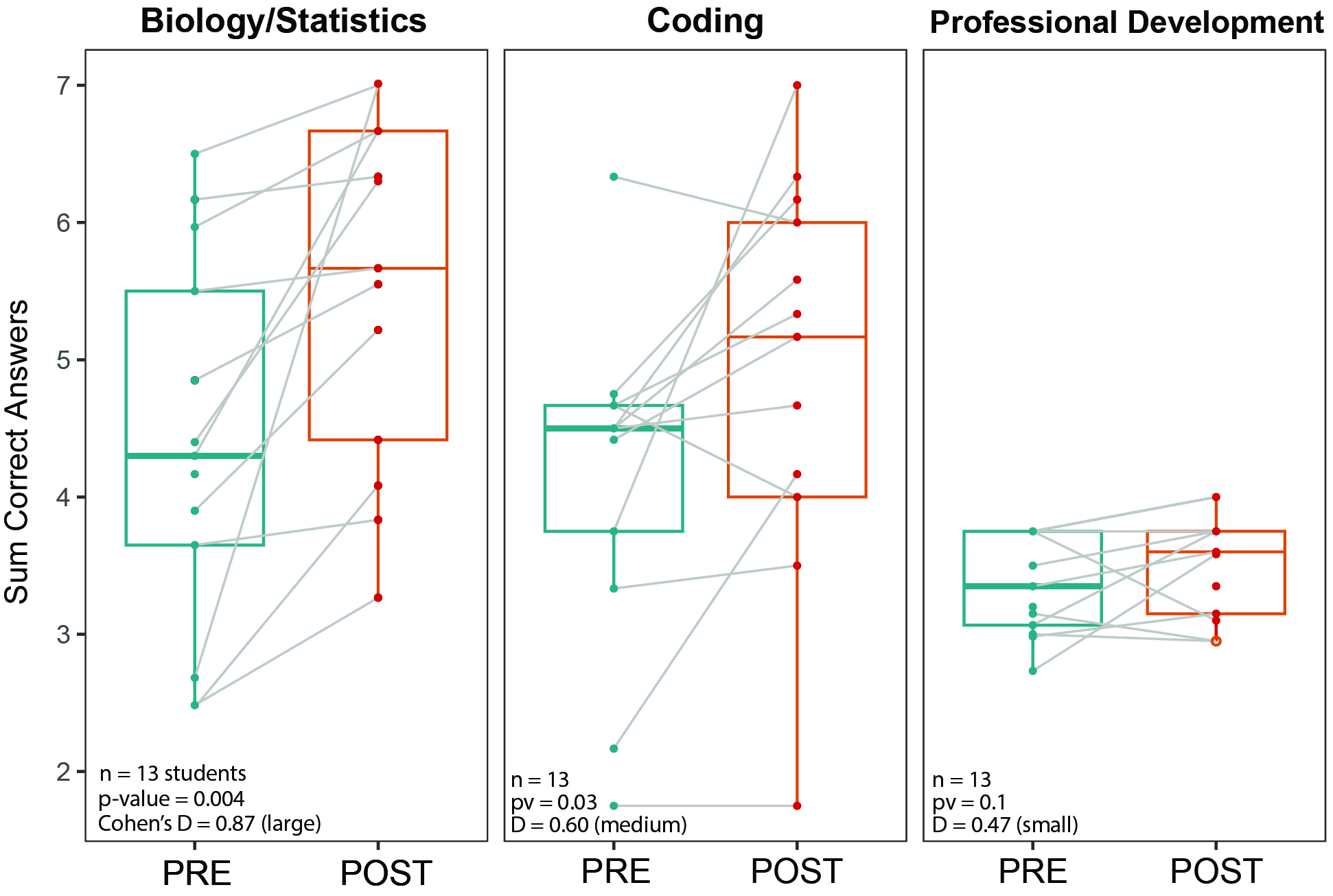

Supplement: S2 Fig — Boxplots depicting each pre-assessment (green) and post-assessment (orange) scores for all questions divided by topic for CURE Iteration 1: Biology/Statistics, Coding, and Professional Development. (TIF) [file pcbi.1012384.s002.tif]

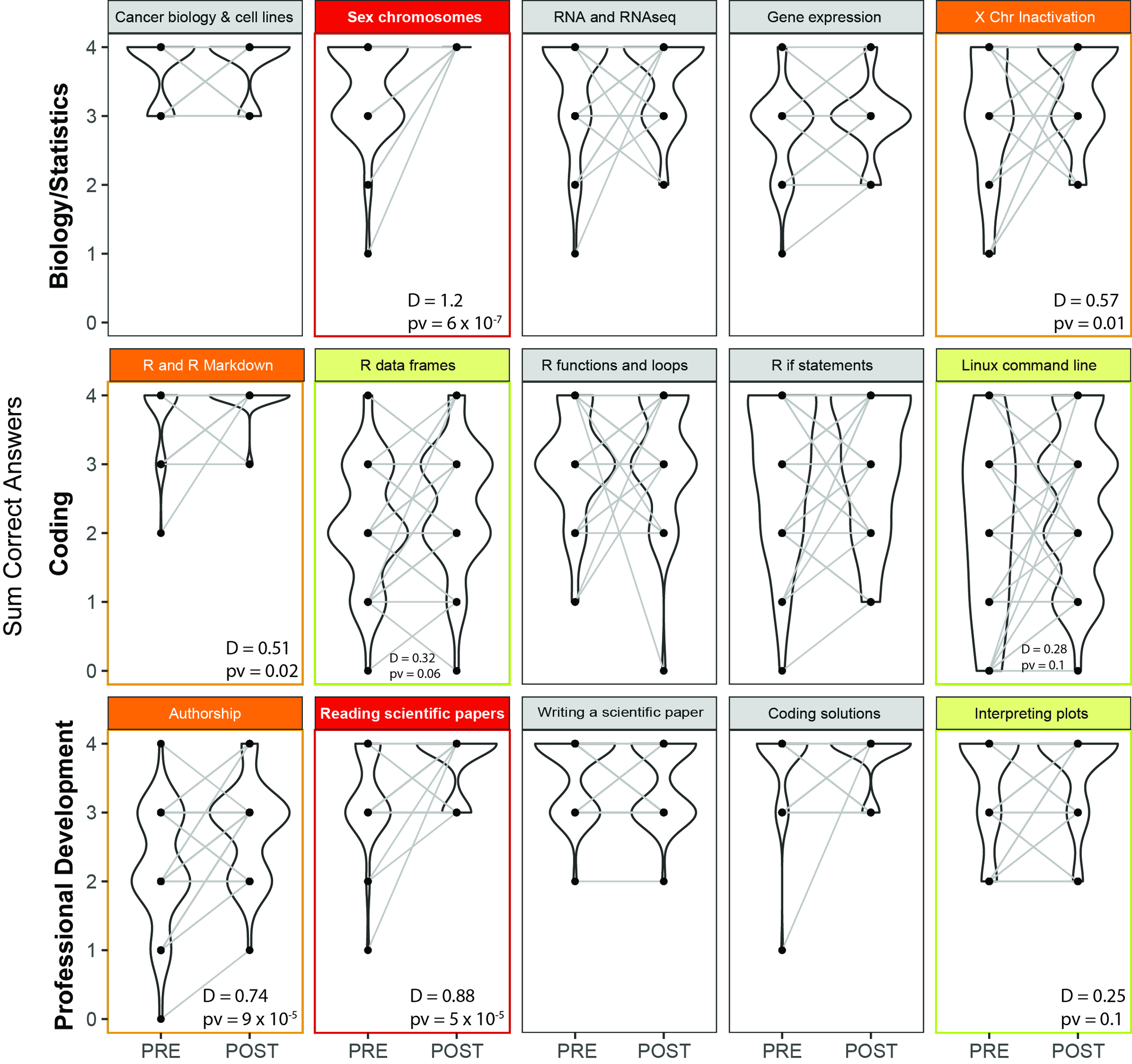

Supplement: S3 Fig — Violin plots depicting each pre-assessment (green) and post-assessment (orange) scores for all questions divided by subtopic for CURE Iteration 2. Subtopics showing paired t test p-value less than 0.01 are highlighted in red, between 0.01 and 0.05 in orange, and between 0.05 and 0.1 in yellow. (TIF) [file pcbi.1012384.s003.tif]

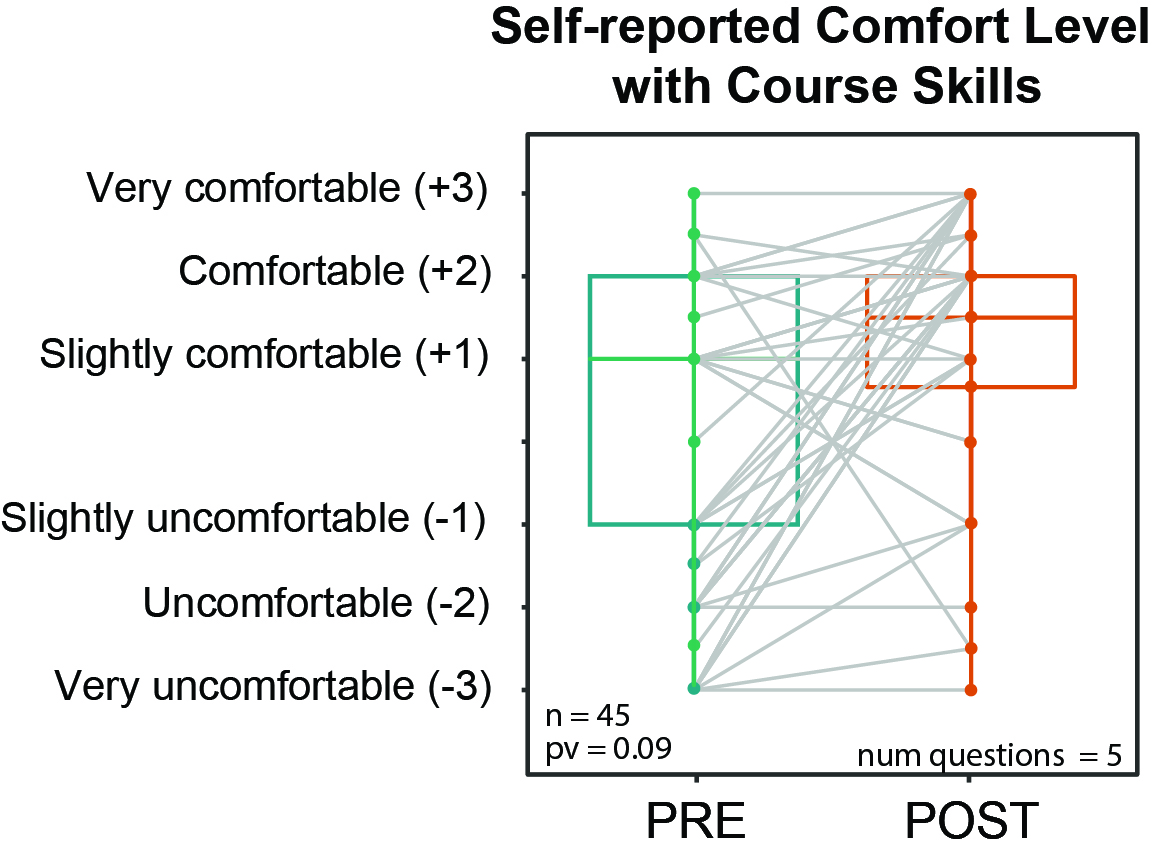

Supplement: S4 Fig — Students were asked to rate their comfort level on a scale from very uncomfortable to very comfortable (6 options total, responses assigned numerical values between −3 and +3) for skills used throughout the course: programming in R, reading and writing scientific papers, asking questions about coding in a class setting, and using command line programming in a Linux environment. Boxplots depicting each pre-assessment (green) and post-assessment (orange) scores for all 5 questions for all students are shown with a paired t test p-value showing the statistical significance of the improvement after the completion of the CURE. (TIF) [file pcbi.1012384.s004.tif]

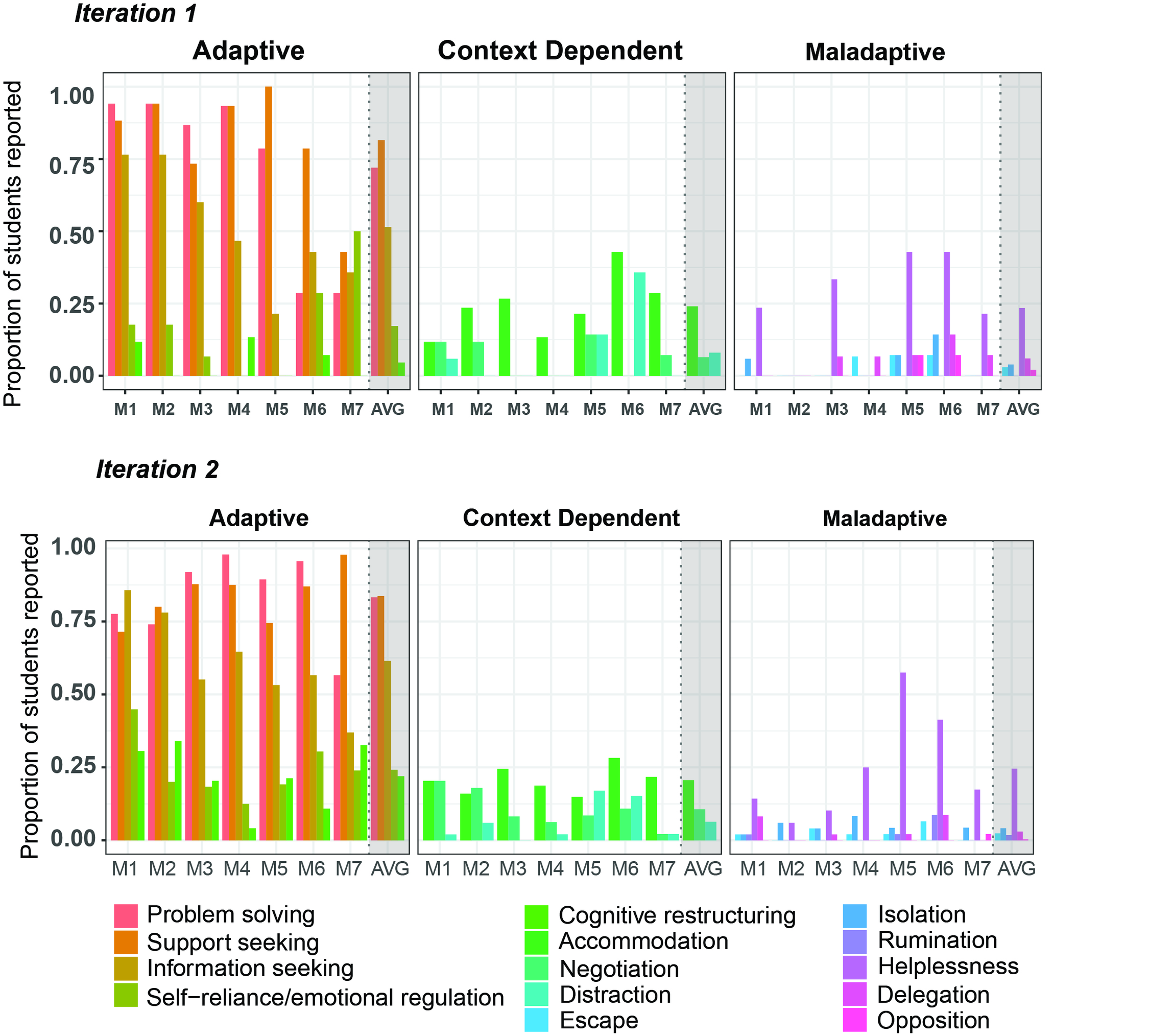

Supplement: S5 Fig — Proportion of students reporting various coping strategies to overcome challenges encountered during genomics research. Responses for progress reports for each module were categorized as adaptive, maladaptive, or those that could be either depending on context. Adaptive themes include problem solving (red), support seeking (orange), information seeking (gold), self-reliance/emotional regulation (olive), and cognitive restructuring (green). Maladaptive themes include escape (light blue), isolation (blue), rumination (purple), helplessness (lilac), delegation (fuchsia), and opposition (pink). (TIF) [file pcbi.1012384.s005.tif]
