# Supplementary material for: Design and implementation of an asynchronous online course-based undergraduate research experience (CURE) in computational genomics
Source: PLoS Comput Biol. 2024 Sep 12;20(9):e1012384. doi: 10.1371/journal.pcbi.1012384 (PMC11392228; doi:10.1371/journal.pcbi.1012384)
Supplement: S1 Methods — (DOCX) [file pcbi.1012384.s006.docx]

# Supplementary Methods

**Github repository for learning materials.** All learning materials, student assessments, and code for analyzing student assessment scores are available in our Github repository (<https://github.com/SexChrLab/CURES>).

**Research Project for Iteration 1 of online genomics CURE.** In a previously published study [(1)](https://paperpile.com/c/oqZAxb/AJkoP), the instructors of this CURE had previously identified genes that were differentially expressed between male and female placentas from full-term pregnancies using a well-established analysis workflow: trimming of low quality RNA sequencing reads and the limma-voom pipeline [(2)](https://paperpile.com/c/oqZAxb/ERpy) for differentially expression of genes between two groups (males and females; <https://github.com/SexChrLab/Placenta_Sex_Diff>). The parameters chosen for trimming adapter sequences and low-quality RNA sequencing reads were determined using experiences of the lead investigators of the study, but literature review showed a variety of thresholds for what was considered to be low quality for reads and recommendations for the effect of trimming on differential gene expression are both limited and contradictory [(3–6)](https://paperpile.com/c/oqZAxb/Fue0j+VZCGU+iH3lE+W675V). The instructors devised a course that would allow students to test a range of values for the ‘trimq’ and ‘minlen’ trimming parameters implemented in the bbduk trimming software [(7)](https://paperpile.com/c/oqZAxb/htuC), the software used in the original publication, to test whether and how the sex differential expression profile was affected in the placenta tissues.

**Research Project for Iteration 2 of online genomics CURE.** Although there is a known sex bias in cancer, sex chromosomes are typically excluded from genomic analyses because of unique analytical challenges associated with their sequence and regulation. Sex chromosome genes include tumor suppressors, epigenetic regulators, immune genes, and more which can impact cancer progression and drug response. A common resource used in cancer research is the Cancer Cell Line Encyclopedia (CCLE), which contains 947 human cancer cell lines used as model systems for studying the biology and treatment of cancer. In this iteration of the CURE, students inferred the sex chromosome complement using the expression of genes on the X and Y chromosomes across these cell lines. We expected the expression of X and Y chromosome genes to match the expected sex chromosome complement given the reported sex of the patient (e.g., typically XX genotype in cell lines from female patients and XY for male patients). Instead we found discordance between the reported sex of the patient and the inferred sex chromosome complement in approximately half of the samples. Determining and comparing the sex chromosome complement is important for assessing how well cell line models recapitulate primary tumors and may reveal molecular underpinnings of sex differences in cancer.

**Data used for the research project.** For the first iteration of the CURE, previously published RNA sequencing data from placenta samples taken at full-term deliveries were the raw reads [(1)](https://paperpile.com/c/oqZAxb/AJkoP). Only the first of two batches of samples collected was used to avoid the need to correct for batch effect, giving 10 placentas from female-bearing pregnancies and 12 placentas from male-bearing pregnancies. Each placenta was sampled twice and each sample was processed for bulk RNA sequencing; gene expression for samples from the same placenta was summed. This was used to address the research question of how robust the published sex differential gene expression profile was to changes in trimming parameters. For the second iteration of the CURE, RNA sequencing gene expression for the Cancer Cell Line Encyclopedia (CCLE) was downloaded from the project’s public data portal (<https://depmap.org/portal/download/all/>, CCLE 2019 dataset, CCLE_RNAseq_genes_counts_20180929.gct.gz) [(8)](https://paperpile.com/c/oqZAxb/xz1G). The data table was converted to a comma-separated value (csv) table by removing the first two rows for easy loading into data frames in R.

**CURE (course-based undergraduate research experience) design and notes on creating custom learning materials.** CUREs can be effectively developed using a “backwards design” in which a research question and appropriate experimental plan are determined and then learning objectives of the course are derived to fit that research plan [(9)](https://paperpile.com/c/oqZAxb/NxktJ). For custom learning materials, the instruction team surveyed publicly available videos, primary literature, and publicly available descriptions and created custom text, figures, and videos to create background materials specific to the chosen research project. To minimize cognitive load and maintain subject relevancy, videos were generally kept to under five minutes long. Shorter videos focused on specific topics make it possible to arrange or update specific topics to customize learning materials for future CUREs. Learning pages were written in short sections so that it was easier for students to complete them in chunks to ease scheduling and instructors to write them in chunks and collaboratively. When possible, instructors tried to make chunks evergreen so that they can be used across many CURE projects.

**Intended audience and prerequisite knowledge.** The CURE was open to students who completed the prerequisite course (BIO 439: Computing for Research) and were enrolled through Arizona State University Online. This is a fully online course with no in-person components. The prerequisite course introduced the students to using genomics software tools in a Linux/Unix shell environment to learn, apply, and write code to process high-throughput sequencing data. The CURE assumed no prior experience with reading or writing publications; formal training on this is provided in the Professional Development sections of the module learning pages, all available for viewing in the Github repository for the online genomics CURE.

**Accessibility**. Learning pages and software were at no added cost to students as project-specific materials were authored by the instruction team and from publicly-available resources. Time estimates were provided separately for each section of each learning module to help students plan their work time. To reduce the need for expensive personal computers, students were strongly encouraged to use the RStudio Server offered by the institution’s high performance computing cluster which reduces the need to store large data files and bulky software packages on local drives. Programming was done with RStudio, a freely available, cross-platform coding environment which the students can continue using through the institution’s high-performance biocomputing cluster if they continue as lab members after course completion, or can download a local copy for their own use off of the cluster. The instructional team aimed to increase accessibility and inclusion when designing learning materials for each module. Communication considerations were at the forefront of the course design and asynchronously leveraged direct messaging platforms (Slack), coordinated video conferences with transcriptions and recordings available, and Canvas-integrated tools such as announcements and email. All videos were downloaded without advertisements and fully transcribed by the instruction team to provide students with a text alternative. This included making sure all pre-recorded videos had transcripts that had the correct scientific jargon and that all learning materials could be accessed for free at any time and place. The class was initially polled for availability to help choose times where the most students could attend optional real-time meetings. All meetings were conducted over Zoom, allowing participants from any location around the world and were recorded with live transcription to increase accessibility. Shared research hours were offered in the evenings and weekends to accommodate students that could not attend at traditional work hours. Arizona State University pays for the full suite of Slack and Zoom capabilities and uses features in Canvas learning management system to automatically create a Slack channel for each class. In cases where the institution does not provide these full versions, both Slack and Zoom have free versions, but if the limitations make it hard to use for the class needs, alternative software such as Discord and Google Meets may serve these needs at a lower monetary cost. Similarly, Arizona State University provides Perusall as an external application that can be used in Canvas for all students, so we used it to post publications for group reading and commenting. External applications for web browsers for shared PDF annotation can be found online at a low cost or the publication can be posted in Google Drive as a document for students to add comments if Perusall is not available through your institution.

**Instructor instructions.** Expectations of the lead faculty instructor were to work with the other course instructors on developing a research project of interest to the field. All course instructors were expected to be knowledgeable about the research project and guide the students through interpreting and contextualizing their findings, but not expected to know the answer to every question any student asked. The nature of a CURE being based on a novel research question meant that there would be questions that no one knew the answer to. Instructors were expected to help troubleshoot code students developed for the research project and use their expertise to show students how to read related publications or interrogate the data further. Specific instructors were expected to grade assignments and progress reports with predetermined rubrics as a way of monitoring progress on the research project, while other instructors were expected to help answer questions on Slack and meet with students during shared research hours. Instructors were aware that fully-online students have shown the same amount of interest in conducting research as their on-campus peers, but have reported a significantly lower awareness of accessible opportunities [(10,11)](https://paperpile.com/c/oqZAxb/lroUL+Scyn9). This may be attributed to the fact that most research experiences, including CUREs, published up to the time of this study were formatted for in-person or hybrid experiences rather than for asynchronous, online students. Instructors reviewed some notable exceptions of courses that transitioned to online and remote learning in response to the COVID-19 pandemic [(12)](https://paperpile.com/c/oqZAxb/ydjDo). Increasing and embedding online research into course schedules can expand access to these learning benefits and continuously improve research programs to adapt to the growing population of online learners, so instructors were educated on the unique challenges that this student population can face.

**Student instructions.** Large scale survey data has shown that undergraduate research experiences increase interest in STEM careers, gains in research skills, and the likelihood that students will pursue graduate degrees [(13–16)](https://paperpile.com/c/oqZAxb/jsK0o+HCN1M+CbbRm+TWKen). While these historically have taken the form of apprenticeships where students work in a faculty member’s lab, there are too few research opportunities for all undergraduate biology students to be able to participate [(17)](https://paperpile.com/c/oqZAxb/FyYra). Students who participate in CUREs can be familiarized with the cultural norms of scientific research, learn to cope with scientific challenges, and be better prepared for future research projects and graduate school [(17–19)](https://paperpile.com/c/oqZAxb/FyYra+qWJql+ZotVW). Students who develop skills in computation and programming are more prepared to answer biological research questions and benefit from the high demand of these skills in the STEM industry [(18,20,21)](https://paperpile.com/c/oqZAxb/Ucex9+qWJql+6aUUM). To set the expectations for the students, the lead instructor began the course by recording an introductory video where she stated that the CURE would guide students through working on a real research project and that the instructors did not know what the outcome would be in advance. She indicated that the students are being graded on their problem solving and their own dedication to advancing the research goals, not the specific outcome of the analysis. She indicated that it was acceptable for the students to feel challenged for any reason (inexperience with research, programming, newness to the field, etc.), but that the students should reach out to the instructors and other students in the class and take advantage of their resources to address those challenges. Students were encouraged to ask questions on the direct messaging platform Slack so that all students in the class could view the responses and learn together. Students were told that the research project would be continued after the CURE and that there would be opportunities for authorship in future symposium presentations and publications. Students were shown a variety of resources that they could use during and after the CURE. Each student was given an account to the university’s high-performance biocomputing cluster free-of-charge; access could be granted after the CURE if the student continued with the project with the lead instructor as a mentor. They were pointed to various resources for learning how to use the cluster as well as ways to ask the research computing department for help. The CURE was maintained using Canvas, the same education management system that was used throughout the institution for accessing grades, syllabi, etc., so that students would feel comfortable with submitting assignments and receiving feedback during assignment grading. In addition to the research project being conducted, the students were told that their participation in the CURE helps to shape future CUREs. By filling out the pre- and post-assessments and the study consent forms, the students were part of an educational study where the instructors could assess what teaching methods gave rise to the most student learning and which concepts were most likely to harbor misconceptions. This explanation was intended to increase the students’ investment into their participation in a CURE and increase their communication and engagement.

**Guidance for storyboarding figures.** The professional development section of Module 5 walks the student through how to storyboard the results attained throughout the CURE. A storyboard includes an outline of the aims of the research project, which topics need to be included in the introduction and methods, and an ordered list of figures with what results they felt they needed to include to state the overall results of the project. Storyboarding helped the students to understand how pieces of the analysis work together to produce an overall conclusion. It also helped the students to separate what was included in the course as background learning from what was done as part of the experimental design and research plan.

**Implementation of course Slack channels**. When the students were enrolled into the CURE, they were invited to a general Slack channel including all of the students enrolled and all of the instructors. Students were allowed to create direct message groups as needed and instructors created channels for small working groups when they felt it would help to facilitate communication. Tips for posting code with proper formatting and sufficient background information were included in the professional development section of the learning pages, along with tips on how to effectively search for coding solutions with internet search engines. Announcements made on Slack were also sent by email, but Slack allowed students to get fast responses from other students and instructors. Some troubleshooting issues were more appropriate for the institution’s research computing staff; in those cases, students were directed to send their Slack message to the research computing help desk Slack channel. At the time of this study, there were no grading options between Canvas and Slack available. During the CURE, Slack was used frequently to communicate with the students and using Slack counted as communication with the class when grading the weekly progress reports. Many students wrote that they got helpful advice from the class Slack channel when they ran into problems. Students mostly posted about coding problems, but also posted questions about the concepts in the learning materials and even shared helpful articles they found while searching for more information and even fun and interesting resources about general science. Instructors tried to respond very quickly to the students; one instructor in the team was typically able to respond within 30 minutes so that made Slack more attractive to students as the course went on. After the CURE was complete, instructors pulled analytics data from Slack to observe trends in engagement. In the first iteration of the CURE (which had a small class size of 13 students), the number of Slack messages posted showed positive correlation with normalized learning gains but in the second iteration (larger class size of 45), we observed more students that were able to increase their course assessment scores without engaging in the class Slack channel as well as a few students who posted a high number of Slack messages but did not see a rise in course assessment scores. The instructors also noticed that some students posted a lot of questions to Slack if they were having coding issues which might reflect those students having a harder time understanding the course material and assignments (which would lead to lower increases in overall assessment scores). Some students from the second iteration (with a larger class size) reported that they felt nervous posting questions to Slack because they didn’t know how the other students would respond or that they were more comfortable having direct conversations through lab meetings, small groups, or emails to the instructors. We anticipate that each iteration of the CURE will have a different dynamic based on the specific cohort of students participating, but the instructors are actively investigating tools for online students to communicate asynchronously and create a sense of community as they learn.

**Implementation of asynchronous online journal club using Perusall**. Perusall social annotation platform was connected to the course Canvas shell as an external application. The 5 publications for journal club were chosen based primarily on relevance to the research project, such as the publication that talked about how the data being analyzed was collected and the original aims of the consortium that collected it. Instructors also included one review article so that students would get experience reading a different type of publication. Learning materials were presented to the students to give them techniques for reading publications quickly and efficiently to retain the most relevant information. The publications chosen for journal club were downloaded as a PDF and posted as an assignment in the module it was meant to be part of. Students were presented with a rubric and Perusall was set up to pass back scores that were put toward a small portion of their final grade. Students made comments that were visible to the whole class as they read the paper, which included questions on parts they felt were confusing, answers on parts they understood well, and ways the research related to knowledge they had from other courses they had taken and concepts they were interested in. Instructors read the papers along with them and were able to tag specific students when they answered questions or shared their own reflections. Students told the instructors that they liked how reading felt more engaging when presented in this way.

**Implementation of pre- and post- learning assessments.** The results from the pre- and post-assessment included only first attempts at the examination and students who completed both assessments were evaluated for learning gains. Students received one attempt to complete the pre-assessment and would receive full credit for the assignment regardless of score. To reduce anxiety levels, there was no time limit on test completion and no video proctoring [(22)](https://paperpile.com/c/oqZAxb/Q7T1), however, a submission was required to move on to subsequent modules. Student pre-assessment and post-assessment results were exported from the Canvas learning management system (LMS) where they were administered and graded. For the iteration 1 of the CURE, questions written to assess scientific learning objectives were given in check-all-that-apply, matching, and multiple-choice and student experience questions were given in Likert scales. To ensure students were getting the correct answers for understanding specific concepts, the check-all-that-apply and matching questions had varying amounts of distractors and answers to be selected, ranging in five to six total options for most questions. The LMS only issued full credit for check-all-that-apply questions that had every selection correct, and deducted points for any distractor selection. For the purpose of this analysis, each question was worth 1.00-point total, and scores ranged depending on the number of options, correct answers, and distractors available per question. For the second iteration of the CURE, scientific learning objectives were assessed with true/false questions, 20 each for Biology/Statistics, Coding, and Professional Development, with 5 subsections of 4 questions each. For analysis, sums of the number of correctly answered questions were compared from before and after the CURE. For student experience questions on the Likert scale, students were asked about comfort level with Linux and command line programming, R programming, reading and writing papers and asking questions about coding in a public forum and asked to select from Very uncomfortable (described numerically as -3), Uncomfortable (-2), Slightly uncomfortable (-1), Slightly comfortable (1), Comfortable (2), or Very comfortable (3). For questions about level of programming skills in any programming language, students were ask to select No experience (0), Beginner (1), Advanced beginner (2), Competent (3), Proficient (4), or Expert (5). Analysis was done to measure the change in comfort and proficiency after completing the CURE.

**Detection of academic dishonesty in submitted writing assignments.** Since a great deal of the grade for the CURE came from written work from students (weekly progress reports, manuscript, peer review, scientific writing prompts, etc.), it was important for instructors to communicate with each other when a student’s writing sample seemed disconnected from the research aims or if different types of writing samples from the same student seemed suspiciously mismatched. Students were made aware before the course began that they were expected to submit their own work and not steal content from other students or other publicly available works. The Canvas learning management system set for the class automatically provided tools to detect answers from multiple students that were suspiciously similar to each other, but if instructors suspected that writing submitted for grading was written by artificial intelligence chatbot engines, free online AI detector tools were used to validate suspicions of academic dishonesty. In the two iterations of the CURE, only one student’s submissions looked suspicious– their responses were written with appropriate grammar and tone but the actual meaning of the words were referring to another scientific topic. For this student, instructors reviewed submissions made throughout the course and found that the average percent of content likely to have been produced by a chatbot was high for many assignments and was distinctly higher than the average percent for the other students in the class. In future iterations of the CURE, instructors will add specific notes about use of chatbots and other resources to the syllabus.

**Student consent and demographics implementation.**  Student surveys were given before and after the completion of the CURE. The first survey asked for consent to participate in the learning assessment portion of the study, demographic information, primary language spoken, and questions about the general anxiety and perceptions around research. These surveys were created using Qualtrics experience management platform. The link to the pre-CURE survey was included in the course introduction module (Module 0) which was required to be completed before the other modules were made available to the students. The post-CURE survey was opened after the other course modules were opened and made available to the students at the end of Module 7. Results of the survey were not shared until the course was completed so as not to introduce bias during analysis of learning gains.

**Data visualizations.** All figures were created in R (version 4.2.1). Pie charts were made using the standard ‘table’ and ‘pie’ function and violin and box plots were made with ‘ggplot2’ package (version 3.4.4).

**Creation of word clouds to summarize progress report entries.** To create word clouds that summarize the accomplishments and challenges sections of the weekly progress report, responses were entered into the ‘wordcloud’ function in the ‘wordcloud’ package (version 2.6) for R (version 4.2.1). Responses were first converted to all lower case and key words “week”, “module”, and “challenge” were removed because many students used these words to label subsections of their progress report. Word use frequencies were colored with a yellow-to-read palette to show the high frequency words in large size and colored in dark red.

**General trends in coping strategies.** The instruction team searched for known coping strategies in student progress reports to see if there were trends in adaptive or maladaptive strategies in different parts of the CURE (**S5 Fig**). Coping strategies were deemed to be adaptive or maladaptive depending on whether they resolve stressors or prevent resolution respectively based on previous definitions [(17–19)](https://paperpile.com/c/niwsY6/4YlPK+r0k7f+5P7gs). Five strategies were adaptive, including problem solving, support seeking, information seeking, self-reliance, and cognitive restructuring. Some strategies - accommodation, negotiation, and distraction - can be adaptive or maladaptive depending on how the student responded to the stressor. Strategies that are generally maladaptive include escape, rumination, helplessness, delegation, and opposition [(17)](https://paperpile.com/c/niwsY6/4YlPK). Results indicated that adaptive coping strategies were employed throughout the CURE by the students (with problem solving and support seeking reported at an especially high level; **(S5 Fig**). These are the methods that instructors were actively encouraging through all modes of communication and learning material. Maladaptive coping strategies increased towards the later modules of the course, with students reporting feeling pressure to finish final projects in all of the other courses taken that semester as well as personal challenges. Time management issues frequently reported throughout the course fell into the accommodation, negotiation, and distraction categories, and were context-dependent for how much they aided or detracted on students progressing on research aims. In some cases, students figured out ways to work around other time-consuming problems in their lives, other times they could not do as much of their research assignments because of those issues. Some students reported maladaptive coping towards the later portion of the course, reporting confusion, low confidence, or concern that their conclusions were not justified as they attempted to interpret results while writing the manuscript (particularly Modules 4, 5, and 6) (**S5 Fig**). Lessons from analyzing coping strategies from progress reports can be used to provide guidance and encouragement for students to help them use more adaptive coping strategies and potentially finding ways to adjust the timing and format of the assignments to better suit the students’ needs.

**Selected quotes from student progress reports.** The following quotes from progress reports help to detail the benefit of this CURE to students and describe how and why they struggled at times. Information that could be used to deidentify the students has been removed.

“This class has been an amazing opportunity for me, especially in terms of my understanding of the research process. It wasn't just an academic exercise but an immersive journey that allowed me to move beyond the traditional roles of participant or support. Instead, I felt fully integrated and invested as an integral part of the team actively engaged in the project. I learned to think like a scientist and felt like one.”

“The information presented in this course was also a challenge for me. But I feel like the course built on itself in a logical way that kept me in a zone where I was uncomfortable with what I didn’t know, but that the knowledge was within reach. I feel like this course had me constantly reaching for the next rung on a ladder. A great challenge for sure.”

“But in the end i have reflected on this whole class as an experience i have learned about from learning how to use R studio to becoming better at reading scientific papers to even being able to write my own scientific papers better. Ive also had the valuable experience of being able to meet all of my classmates who have been asking questions and helping each other through Slack. I would say the best experiences or the most i learned was in the coding section i started very new to coding and have ended still pretty new but i understand significantly more then i used to know.”

“I feel more confident now than I have in the entirety of the course regarding my ability use RStudio and how to utilize code to analyze data and come up with results. While I still have a lot to learn regarding this, I feel comfortable knowing that I have a good foundation that will allow me to grow my knowledge without direct instruction from another individual. This course has given me a self confidence in my own abilities to troubleshoot and problem solve with the resources available to me.”

“This week presented a unique set of challenges, primarily centered around the complexity of understanding and coding in R. As a novice in this programming language, I found the intricacies of coding for a complex genomic analysis project initially overwhelming. The pressure to perform well in an unfamiliar coding language, combined with the need to manage time effectively in what felt like a rushed project, added to the learning curve.

The first major challenge was my difficulty in comprehending R coding. To tackle this, I embraced collaborative learning, engaging in discussions with my classmate, [XXX], who provided a clearer understanding of the assignment's objectives. These conversations helped demystify the task and made the coding aspects more approachable. Additionally, I sought guidance from Dr. Plaisier, whose insights were pivotal in aligning my efforts with the expected outcomes, thereby clarifying the exact requirements for the file submission.

Another significant challenge was managing my time efficiently under the pressure of a tight timeframe. I addressed this by prioritizing tasks and breaking down the project into manageable segments, which alleviated the feeling of being rushed and allowed for more efficient progress.

Furthermore, the class Slack channel proved to be an invaluable resource. It not only assisted in understanding the grading process, reducing my stress, but also provided a platform for peer support. By actively participating in Slack discussions, I not only sought assistance but also offered help to others. Sharing my experiences and solutions with peers who might have faced similar issues not only contributed to our collective learning experience but also reinforced my own understanding of the subject matter. This approach of mutual support and collaboration was instrumental in navigating the challenges of the week.”

“This week, first of all I'm still soaring on my accomplishments from writing my first (sort of) manuscript. Truthfully this whole course was very intimidating for me. I'm very proud of my abilities and what I was able to accomplish. I gained so much insight on cancer, genetics, coding, and overall research strategies.”

“In terms of concepts and coding learned, the experience was immensely enlightening. The peer review process of the cumulative manuscripts allowed me to grasp the expected outcomes and standards of our project more clearly. It was a revealing journey into the sex-specific molecular characteristics of cancer, offering insights into how these differences could inform personalized therapeutic strategies. The project, albeit challenging, was instrumental in enhancing my understanding of genomic analysis and its complexities.”

“Successful communication was a cornerstone of my learning experience in this project. A pivotal moment was my discussion with a fellow student, [XXX], which helped clarify the objectives of the week's assignment. This conversation was instrumental in providing a student's perspective on the project, aiding in demystifying the complex task at hand. Additionally, my interactions with Dr. Plaisier were crucial. Speaking with her allowed me to grasp the exact expectations for the file upload, ensuring that my work aligned precisely with the project's requirements.”

“Seeing my classmates' choices and writing styles, as well as their inclusions and omissions, was enlightening. It taught me that there are multiple valid ways to approach the same research problem, each with its unique strengths. Importantly, this experience has taught me the crucial skill of narrowing down choices and testing specific outcomes, rather than attempting to cover too broad a scope. In the past, I tended to aim for generalizations, which often led to oversimplifications and weakened the results of my studies. Now, I understand the importance of focusing on specific variables to avoid inflating results and to make my research more robust. Overall, this week was more about growing as a researcher than just about accomplishing tasks.”

“As an undergrad, I worked in a couple labs [XXX], and I did field work as a [XXX]. But I never felt like I was exposed to where that data went or how it was used. I always felt I was lacking as a biologist. I’ve worked as an instructional assistant [XXX], and I see a distinct lack of computational skills being presented in undergrad courses. The junior college I work at is an unofficial pipeline to [XXX] and a lot of the students I help have medical school as their goal. I’ve been able to share my research with them this semester, and I see a light go on where they had not considered how valuable coding is for biology. My goal is to become a professor at a junior college, and I feel significantly more competent after computing for research and this research course. I’m looking forward to finding ways of introducing coding and data analysis into curriculum for general biology courses. There are so many general education courses students must take to transfer to 4-year colleges, I think it’s grossly outdated that coding is not a requirement for STEM majors.”

# References

1. [Olney KC, Plaisier SB, Phung TN, Silasi M, Perley L, O’Bryan J, et al. Sex differences in early and term placenta are conserved in adult tissues. Biol Sex Differ. 2022 Dec 22;13(1):74.](http://paperpile.com/b/oqZAxb/AJkoP)

2. [Law CW, Chen Y, Shi W, Smyth GK. voom: Precision weights unlock linear model analysis tools for RNA-seq read counts. Genome Biol. 2014 Feb 3;15(2):R29.](http://paperpile.com/b/oqZAxb/ERpy)

3. [Williams CR, Baccarella A, Parrish JZ, Kim CC. Trimming of sequence reads alters RNA-Seq gene expression estimates. BMC Bioinformatics. 2016 Feb 25;17:103.](http://paperpile.com/b/oqZAxb/Fue0j)

4. [Liao Y, Shi W. Read trimming is not required for mapping and quantification of RNA-seq reads at the gene level. NAR Genom Bioinform. 2020 Sep;2(3):lqaa068.](http://paperpile.com/b/oqZAxb/VZCGU)

5. [Macmanes MD. On the optimal trimming of high-throughput mRNA sequence data. Front Genet. 2014 Jan 31;5:13.](http://paperpile.com/b/oqZAxb/iH3lE)

6. [Del Fabbro C, Scalabrin S, Morgante M, Giorgi FM. An extensive evaluation of read trimming effects on Illumina NGS data analysis. PLoS One. 2013 Dec 23;8(12):e85024.](http://paperpile.com/b/oqZAxb/W675V)

7. [Bushnell B, Rood J, Singer E. BBMerge – Accurate paired shotgun read merging via overlap. PLoS One. 2017 Oct 26;12(10):e0185056.](http://paperpile.com/b/oqZAxb/htuC)

8. [Barretina J, Caponigro G, Stransky N, Venkatesan K, Margolin AA, Kim S, et al. The Cancer Cell Line Encyclopedia enables predictive modelling of anticancer drug sensitivity. Nature. 2012 Mar 28;483(7391):603–7.](http://paperpile.com/b/oqZAxb/xz1G)

9. [Cooper KM, Soneral PAG, Brownell SE. Define Your Goals Before You Design a CURE: A Call to Use Backward Design in Planning Course-Based Undergraduate Research Experiences. J Microbiol Biol Educ [Internet]. 2017 Apr;18(2). Available from:](http://paperpile.com/b/oqZAxb/NxktJ) <http://dx.doi.org/10.1128/jmbe.v18i2.1287>

10. [Faulconer EK, Dixon Z, Griffith J, Faulconer L. Perspectives on Undergraduate Research Mentorship: A Comparative Analysis Between Online and Traditional Faculty. Online Journal of Distance Learning Administration. 2020;23(2):1.](http://paperpile.com/b/oqZAxb/lroUL)

11. [Cooper KM, Gin LE, Brownell SE. Diagnosing differences in what Introductory Biology students in a fully online and an in-person biology degree program know and do regarding medical school admission. Adv Physiol Educ. 2019 Jun 1;43(2):221–32.](http://paperpile.com/b/oqZAxb/Scyn9)

12. [DeHaven B, Sato B, Mello J, Hill T, Syed J, Patel R. Bootleg Biology: a Semester-Long CURE Using Wild Yeast to Brew Beer. J Microbiol Biol Educ [Internet]. 2022 Dec;23(3). Available from:](http://paperpile.com/b/oqZAxb/ydjDo) <http://dx.doi.org/10.1128/jmbe.00336-21>

13. [Russell SH, Hancock MP, McCullough J. The pipeline. Benefits of undergraduate research experiences. Science. 2007 Apr 27;316(5824):548–9.](http://paperpile.com/b/oqZAxb/jsK0o)

14. [Lopatto D. Survey of Undergraduate Research Experiences (SURE): first findings. Cell Biol Educ. 2004 Winter;3(4):270–7.](http://paperpile.com/b/oqZAxb/HCN1M)

15. [Lopatto D. Undergraduate research experiences support science career decisions and active learning. CBE Life Sci Educ. 2007 Winter;6(4):297–306.](http://paperpile.com/b/oqZAxb/CbbRm)

16. [Paalman MH. Undergraduate research, education and the future of science. Anat Rec. 2002 Feb 15;269(1):1–2.](http://paperpile.com/b/oqZAxb/TWKen)

17. [Bangera G, Brownell SE. Course-based undergraduate research experiences can make scientific research more inclusive. CBE Life Sci Educ. 2014 Winter;13(4):602–6.](http://paperpile.com/b/oqZAxb/FyYra)

18. [Bennett JA. The CURE for the Typical Bioinformatics Classroom. Front Microbiol. 2020 Aug 12;11:1728.](http://paperpile.com/b/oqZAxb/qWJql)

19. [Gin LE, Rowland AA, Steinwand B, Bruno J, Corwin LA. Students Who Fail to Achieve Predefined Research Goals May Still Experience Many Positive Outcomes as a Result of CURE Participation. CBE Life Sci Educ. 2018 Dec;17(4):ar57.](http://paperpile.com/b/oqZAxb/ZotVW)

20. [Wilson Sayres MA, Hauser C, Sierk M, Robic S, Rosenwald AG, Smith TM, et al. Bioinformatics core competencies for undergraduate life sciences education. PLoS One. 2018 Jun 5;13(6):e0196878.](http://paperpile.com/b/oqZAxb/Ucex9)

21. [Gao L, Guo M. A course-based undergraduate research experience for bioinformatics education in undergraduate students. Biochem Mol Biol Educ. 2023 Mar;51(2):189–99.](http://paperpile.com/b/oqZAxb/6aUUM)

22. [Gin LE, Pais DC, Parrish KD, Brownell SE, Cooper KM. New Online Accommodations Are Not Enough: The Mismatch between Student Needs and Supports Given for Students with Disabilities during the COVID-19 Pandemic. J Microbiol Biol Educ [Internet]. 2022 Apr;23(1). Available from:](http://paperpile.com/b/oqZAxb/Q7T1) <http://dx.doi.org/10.1128/jmbe.00280-21>
